# Integrin Adhesion Complex Organization in Sheep Myometrium Reflects Changing Mechanical Forces during Pregnancy and Postpartum

**DOI:** 10.3390/biology10060508

**Published:** 2021-06-08

**Authors:** Bryan A. McLendon, Avery C. Kramer, Heewon Seo, Fuller W. Bazer, Robert C. Burghardt, Gregory A. Johnson

**Affiliations:** 1 Department of Veterinary Integrative Biosciences, College of Veterinary Medicine and Biomedical Sciences, Texas A&M University, College Station, TX 77843, USA; bmclendon@cvm.tamu.edu (B.A.M.); akramer@cvm.tamu.edu (A.C.K.); hseo@cvm.tamu.edu (H.S.); 2 Department of Animal Science, Texas A&M University, College Station, TX 77843, USA; fbazer@cvm.tamu.edu

**Keywords:** integrin adhesion complex (IAC), mechanosensation/mechanotransduction, myometrium, pregnancy, sheep

## Abstract

**Simple Summary:**

IACs assemble within the sheep myometrium during early-to-mid gestation in response to increased stretch of the uterine wall and continue to increase as pregnancy progresses. Fibronectin (FN1) is important in its ability to attach to IACs in myometrial cells to generate force to sustain powerful contractions during labor. After parturition, IACs are disassembled but the integrin subunits ITGA5 and ITGB1 remain expressed at the protein level at least two weeks postpartum.

**Abstract:**

Cells respond to extracellular mechanical forces through the assembly of integrin adhesion complexes (IACs) that provide a scaffold through which cells sense and transduce responses to those forces. IACs are composed of transmembrane integrin receptors that bind to extracellular matrix (ECM) proteins externally and connect with the actomyosin cytoskeleton internally. Myometrial smooth muscle cells respond to forces that arise due to increases in fetal growth/weight, placental fluid volumes, and blood flow. As a result, the uterus transforms into an organ that can forcefully expel the fetus and placental membranes during parturition. While earlier studies focused on IAC expression in the myometrial compartment of rodents and humans to explore pregnancy-associated responses, the present study examines IAC assembly in ovine myometrium where mechanical forces are expected to be amplified in a manner similar to humans. Results indicate that the ITGA5 and ITGB1 heterodimers associate with the ECM protein FN1 externally, and with VCL and TLN1 internally, to form IACs in myometrial cells during the first trimester of pregnancy. These IACs become increasingly ordered until parturition. This ordered structure is lost by one day postpartum; however, the abundance of the integrin proteins remains elevated for at least two weeks postpartum. Implications of the present study are that sheep are similar to humans regarding the assembly of IACs in the pregnant myometrium and suggest that IACs may form much earlier in human gestation than was previously implied by the rat model. Results highlight the continued value of the sheep model as a flagship gynecological model for understanding parturition in humans.

## 1. Introduction

Throughout pregnancy, the myometrium undergoes hypertrophy in response to the developing fetus and associated placental membranes [1]. This includes cell proliferation, increased ECM production, and remodeling of integrin-focal adhesion (FA) contacts [2]. FAs, a type of integrin adhesion complex (IAC), are consistently seen in cultured cells [3,4], although they are less obvious in vivo.

The onset of parturition is initiated by the fetus and relies on both endocrine and mechanical signaling pathways caused by uterine stretch in the myometrium. In sheep, the endocrine signaling pathway involves a change in the ratio of estrogen to progesterone secretion that primes the uterus for myometrial activation, critical for delivery of the fetus [5]. The simultaneous increase of estrogen and decrease of progesterone increases the contractility of the myometrium, in part, by increasing contraction-associated proteins (including gap junctions, comprised of connexin 43 (Cx43), oxytocin receptor, and others) which allow for direct electrical and chemical communication between the muscle fibers [6]. In addition to endocrine signaling, mechanotransduction by integrins plays an important role in parturition, with mechanical stretch promoting extensive uterine hypertrophy and remodeling in pregnant and postpartum animals [7,8,9].

Integrin activation through binding with the ECM leads to assembly of IACs that relay a variety of signals involved in many cellular processes such as cellular adhesion, migration, and stimulation of numerous signal transduction pathways [10]. Each integrin is a heterodimer that includes an alpha and beta subunit, and the alpha/beta heterodimers specify the ligand-binding abilities of the integrin. For example, the alpha 5 integrin subunit (ITGA5) partners exclusively with the beta 1 integrin subunit (ITGB1), forming the ITGA5/ITGB1 (ITGA5B1) integrin receptor [11]. This is a classic mechanosensory receptor for the matricellular protein fibronectin (FN1). FN1 is a multimodular force-bearing ECM protein that can exhibit a wide range of conformations in the ECM based on substrate rigidity [12]. It is secreted by cells and assembled into a matrix that binds to cell surface integrin receptors, which, under tension, can lead to conformational changes of FN1 into different functional signaling states. The binding of FN1 to ITGA5B1 can promote FN1 fibrillogenesis to provide a dense meshwork of interconnected ECM supporting strong intercellular cohesion [11,12,13,14]. Talin (TLN1) and vinculin (VCL) are interacting cytoplasmic adaptor proteins that connect the ITGA5B1 integrin receptor to the actin cytoskeleton, allowing for communication and mechanotransduction between cells and/or cell-ECM contacts [12,15,16].

In vitro studies revealed that size as well as the specific protein components of IACs are dependent on the rigidity of the ECM and the internal or external mechanical forces applied to the integrin-ECM complex [17,18]. IACs organize into linear strands called dense plaques within the smooth muscle cells of hollow organs [19,20]. Smooth muscle IACs are present in many organs, including the smooth muscle surrounding blood vessels, to create a cohesive functional syncytium that can coordinate contractility of the organ or vessel wall [2]. Studies in rats showed that ITGA5 integrin gene and protein expression increase within the myometrium and incorporate into IACs during late pregnancy and labor [2]. Similar observations have been made for multiple integrins in the nonlaboring and laboring myometrium of women [21]. The ITGA5 integrin and multiple adaptor proteins have been localized in the myometrium of sheep during gestation [22]. However, less is known about IACs located within the sheep myometrium during the pre- and postpartum periods.

Fetal size is similar between sheep and humans. The uterus of both species undergoes significant growth during late pregnancy resulting in uterine stretch and parturition requires similar coordinated smooth muscle contractions. The procurement of myometrial tissues from women over the course of pregnancy is problematic for ethical reasons. The temporal assembly of IACs within the myometrium throughout pregnancy has only been reported for the rat, a species with far smaller fetuses and proportionately less stretch of the uterine wall [2]. We hypothesized that the uterus of sheep develops the mechanical strength to expel the fetus and placenta at parturition by assembling highly ordered IACs between myometrial cells during late pregnancy. These IACs subsequently disassemble after parturition. Therefore, we examined the spatial expression of ITGA5, ITGB1, FN1, TLN1, and VCL proteins, all components of IACs, in the myometrium of sheep throughout gestation and after parturition. We also examined the same IAC components in myometrial cells grown on either uncoated or FN1-coated rigid substrates. Results of the present study indicate that sheep are similar to humans regarding the assembly of IACs in the pregnant myometrium and suggest that IACs may form much earlier in human gestation than was previously implied by the rat model. Results highlight the continued value of the sheep model as a flagship gynecological model for understanding parturition in humans [5].

## 2. Materials and Methods

### 2.1. Animals and Tissue Collection

All animal experiments complied with the Guide for Care and Use of Agricultural Animals and were approved by the Institutional Agricultural Animal Care and Use Committee of Texas A&M University (AUP IACUC 2012-161). Ewes (*Ovis aries*) were observed daily for estrus in the presence of vasectomized rams. Cyclic ewes were ovariohysterectomized on Day 15 of the estrous cycle (*n* = 4). Ewes were mated to intact rams three times at 12 h -intervals beginning at the onset of estrus (Day 0). Pregnant ewes were ovariohysterectomized on either Day 15, 40, 80, 120, or 140 of pregnancy and on Day 1, 7, or 14 postpartum (*n* = 4 ewes/day). Myometrial tissue was dissected from the endometrium and stored at −80 °C until processed for Western blot analyses. In addition, several sections (~1.5 cm) from the middle of each uterine horn were embedded in Tissue-Tek Optimal Cutting Temperature (OCT) Compound (Miles, Oneonta, NY, USA), frozen in liquid nitrogen vapor, and stored at −80 °C until processed for immunofluorescence analyses.

### 2.2. Cell Culture

For in vitro studies, we utilized the human myometrial cell line PHM1-41, which was obtained from Dr. Barbara Sanborn and derived from term-pregnant human myometrium (patients not in labor) using a protocol that had received prior institutional approval and immortalized using a vector expressing human papillomavirus E6 and E7 proteins [23]. Cells were cultured in DMEM-F12 plus 10% fetal calf serum.

### 2.3. Western Blot Analyses

Myometrial samples were homogenized in lysis buffer (1% Triton X-100, 0.5% Nonidet P-40, 150 mM NaCl, 10 mM Tris, 1 mM EDTA, 0.1 mM EGTA, 0.2 mM Na_3_VO_4_, 0.2 mM phenylmethylsulfonyl fluoride, 50 mM NaF, 30 mM Na_4_P_2_O_7_, 1 mg/mL leupeptin, 1 mg/mL pepstatin) at a ratio of 1 g tissue per 5 mL buffer. Cellular debris was cleared by centrifugation (12,000× *g*, 15 min, 4 °C). The protein concentration of the supernatant was determined using a Bradford protein assay (Bio-Rad Laboratories, Hercules, CA, USA) with BSA as the standard. Western blot analyses were performed as described previously [24]. Briefly, proteins (10 µg) were denatured in Laemmli buffer, separated on 8% SDS-PAGE gels, and transferred to nitrocellulose. Blots were blocked in 5% nonfat milk/TBST (Tris-buffered saline, 0.1% Tween-20) at room temperature for 1 h, incubated with either rabbit anti-ITGA5 (#AB1928; 1:1000) or rabbit anti-ITGB1 (#AB1952; 1:1000) from Chemicon (Temecula, CA, USA) [22], or normal rabbit IgG (Sigma Aldrich, St. Louis, MO, USA; 1:1000) in 2% nonfat milk/TBST overnight at 4 °C. Blots were then rinsed three times for 10 min each with TBST at room temperature, incubated with goat anti-rabbit IgG horseradish peroxidase conjugate (1:20,000 dilution of 1 mg/mL stock; Kirkegaard & Perry Laboratories, Bethesda, MD, USA), and then rinsed three times for 10 min each with TBST. Immunoreactive proteins were detected using enhanced chemiluminescence (SuperSignal West Pico Luminol System, Pierce Chemical Co., Rockford, IL, USA) according to the manufacturer’s recommendations using a FluorChem IS-8800 120 imager (Alpha Innotech, San Leandro, CA, USA). Blots were quantified using AlphaEase FC software (Alpha Innotech).

### 2.4. Immunofluorescence Analyses

For immunofluorescence staining, primary antibodies included rabbit anti-ITGA5 (#AB1928; 1:200), rabbit anti-ITGB1 (#AB1952; 1:200), and mouse anti-FN1 (#MAB88916; 1:200) from Chemicon (Temecula, CA), and mouse anti-talin clone 8d4 (#T3287; 1:100), mouse anti-vinculin clone hVN-1 (#V9131; 1:200), normal rabbit IgG (#15006; 1:200), and normal mouse IgG (#15381; 1:100 or 1:200) from Sigma Aldrich (St. Louis, MO, USA). The secondary antibodies included fluorescein-conjugated goat anti-rabbit IgG (Chemicon; 1:250), fluorescein-conjugated goat anti-mouse IgG (Zymed, San Francisco, CA, USA; 1:250), and Texas Red-conjugated goat anti-rabbit IgG (Molecular Probes, Eugene, OR, USA).

The immunostaining of frozen sections of myometrium allowed for the localization of ITGA5, ITGB1, FN1, TLN1, and VCL proteins as previously described [25]. Briefly, frozen sections (~10 μm) of uterine wall, including the myometrium, were cut with a cryostat (Hacker-BrightOTF, Hacker Instruments, Inc., Winnsboro, SC, USA) and mounted on Superfrost/Plus microscope slides (Fisher Scientific, Pittsburgh, PA, USA). Sections were fixed in −20 °C methanol for 10 min, permeabilized at room temperature with 0.3% Tween-20 in 0.02 M PBS (rinse solution), and blocked in 10% normal goat serum for 1 h at room temperature. Sections were then dipped in rinse solution at room temperature, incubated overnight at 4 °C with each primary antibody, and detected with fluorescein-conjugated secondary antibody. Slides were then overlaid with a cover-glass and Prolong antifade mounting reagent (Molecular Probes, Eugene, OR, USA). Negative controls included the appropriate normal rabbit or mouse IgG at the same concentration as primary antibodies. For co-localization of proteins, frozen sections were prepared as described above.

The immunostaining of cultured human myometrial PHM1-41 cells involved seeding cells on Lab-Tek glass chambered slides that were either untreated or pretreated overnight with 50 µg/mL of human fibronectin 120 kDa α chymotryptic fragment containing the cell attachment region (Sigma Aldrich, St. Louis, MO, USA). Myometrial cells were then added and allowed to attach in DMEM-F12 plus 10% fetal calf serum. Cells were then washed in PBS, chambers were removed leaving a silicone gasket surrounding each well, and fixed in −20 °C methanol for 10 min. The immunofluorescence co-localization of proteins was then performed as described previously [26]. After washing with PBS containing 0.3% (vol/vol) Tween-20, slides were incubated overnight at 4 °C with initial primary antibody (either anti-ITGA5, -ITGB1, -FN1, or -VCL IgG at dilutions described above). Following three washes in 4 °C rinsing solution for 10 min each, slides were incubated with initial secondary antibody (either FITC- or Texas Red-conjugated anti-mouse IgG at dilutions described above) for 4 h at room temperature and washed in 4 °C rinsing solution 6 times for 10 min each. Slides were then incubated overnight at 4 °C with the second primary antibody (either anti-ITGA5, -ITGB1, -FN1, or -VCL IgG at dilutions described above). Following six washes in 4 °C rinsing solution for 10 min each, slides were incubated with 2 μg/mL of the second secondary antibody (either FITC- or Texas Red-conjugated anti-rabbit IgG at the dilutions described above) for 2 h at 4 °C, washed 6 times in 4 °C rinsing solution for 10 min each, and dipped in distilled-deionized H_2_O. Gaskets were removed and antifade mounting reagent was added prior to overlaying a coverslip. Immunofluorescence images were acquired using an Axioplan 2 microscope (Carl Zeiss, Thornwood, NY, USA) interfaced with an Axioplan HR digital camera.

### 2.5. Statistical Analyses

Data were subjected to least-squares ANOVA using the general linear models procedures of the Statistical Analysis System (SAS, Cary, NC, USA). The abundances of ITGA5 and ITGB1 proteins were determined by Western blotting and evaluated within each blot to control for any differences in exposure times. All tests of significance were performed using the appropriate error terms according to the expectation of the mean squares for error. Data are presented as least-squares means with overall standard errors.

## 3. Results

### 3.1. ITGA5 and ITGB1 Proteins Increase in the Myometrium during Mid-Gestation and Are Maintained Postpartum

**Figure 1:**
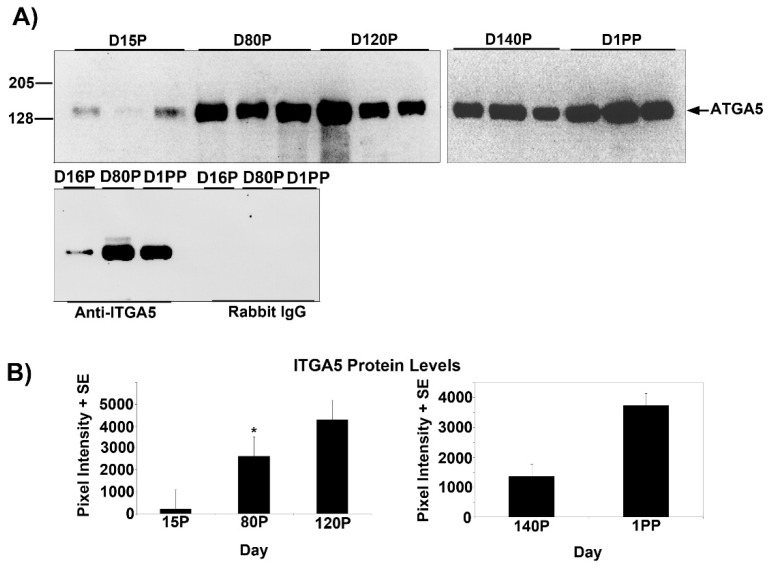
(**A**) Western blot detection of the alpha 5 integrin (ITGA5, 8% 1D-PAGE) in ovine myometrial extracts (10 μg/lane) from different Days of pregnancy (P) or postpartum (PP). Each lane represents a sample from a different ewe. Immunoreactive proteins were detected using a rabbit anti-ITGA5 IgG or irrelevant rabbit IgG. The positions of pre-stained molecular weight standards are indicated on the left of the gels. (**B**) Quantification of total ITGA5 protein in myometrial tissue as detected by Western blotting. ITGA5 protein increased on Day 80 of gestation, and expression remained elevated through Day 14 postpartum, as indicated by the *asterisk* (*p* < 0.05).

**Figure 2:**
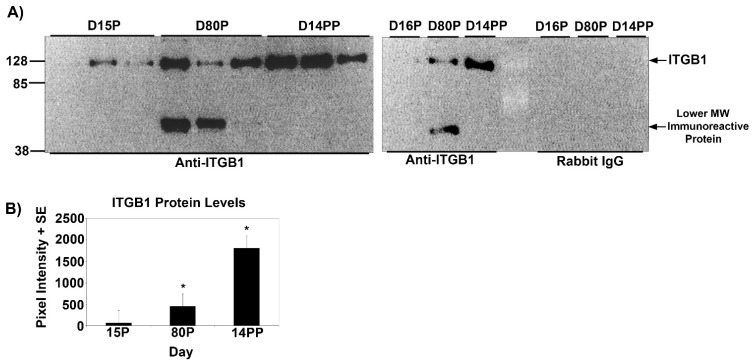
(**A**) Western blot detection of the beta 1 integrin (ITGB1, 8% 1D-PAGE) in ovine myometrial extracts (10 μg/lane) from different Days of pregnancy (P) or postpartum (PP). Each lane represents a sample from a different ewe. Immunoreactive proteins were detected using a rabbit anti-ITGB1 IgG or irrelevant rabbit IgG. The positions of pre-stained molecular weight standards are indicated on the left of the gels. (**B**) Quantification of total ITGB1 protein in myometrial tissue as detected by Western blotting. ITGB1 protein increased on Day 80 of gestation, and expression remained elevated through Day 14 postpartum, as indicated by the *asterisk* (*p* < 0.05).

Temporal changes in the expression of ITGA5 and ITGB1 proteins in the myometrium of pregnant and postpartum ewes were determined using Western blotting (Figure 1 and Figure 2). ITGA5 migrated to ~130 kDa on all Days examined, whereas ITGB5 migrated to ~128 kDa on all Days examined; however, a lower MW band was also observed on Day 80 of gestation. The lower molecular weight form has not been reported in the literature but is observed for the product quality control Western blots for the anti-ITGB5 IgGs from some companies. At present, we do not speculate on the identity of this immunoreactive protein. On Day 15 of gestation, ITGA5 and ITGB1 proteins were expressed at low levels. However, on Day 80, abundance of ITGA5 and ITGB1 proteins in the myometrium increased significantly compared with Day 15 and abundant levels of ITGA5 and ITGB1 proteins were maintained for the remainder of pregnancy and through Day 14 postpartum (Figure 1 and Figure 2).

### 3.2. ITGA5 and ITGB1 Proteins Assemble into Longitudinally Oriented IACs at the Surface of Myometrial Cells during Late Pregnancy That Disperse Postpartum

**Figure 3:**
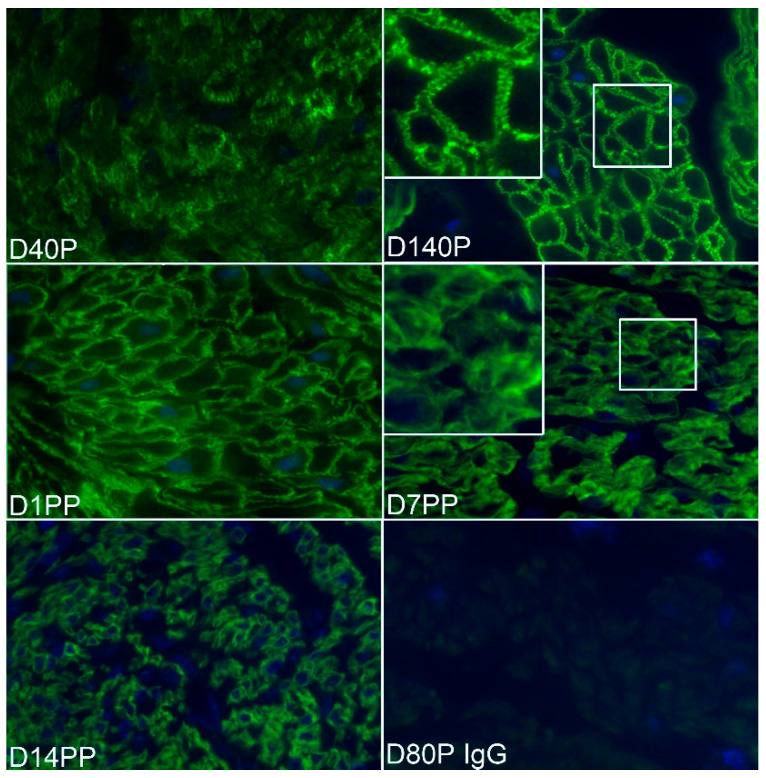
Immunostaining for the alpha 5 integrin (ITGA5) in the myometrium of ewes on Days (D) 40 and 140 of pregnancy (P) and Days 1, 7, and 14 postpartum (PP). Note the linear strands of IACs that appear as regularly distributed spots at the myometrial cell surface present in some cells on Day 40, which are present and highly ordered on all myometrial cells by Day 140 of pregnancy. These highly ordered IACs are no longer present in the myometrium of postpartum ewes. For comparison, insets provide surface views of myometrial cells during late pregnancy and postpartum. An irrelevant rabbit IgG served as a negative control. The width of each field is 140 μm; the inset is 25 μm.

**Figure 4:**
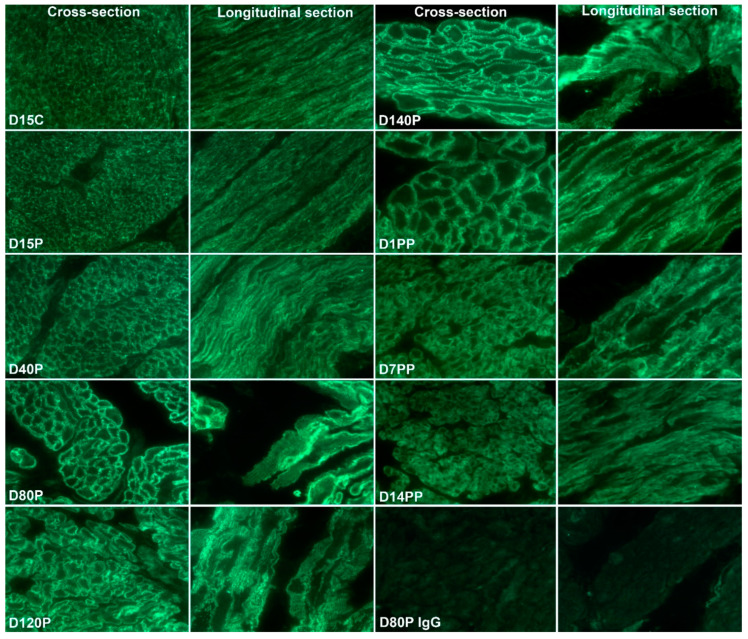
Immunostaining for beta 1 integrin (ITGB1) in the myometrium of ewes on Day (D) 15 of the estrous cycle (C), Days 15, 40, 80, 120, and 140 of pregnancy (P), and Days 1, 7, and 14 postpartum (PP) is shown. Note the linear strands of IACs that appear as regularly distributed spots at the myometrial cell surface present in some cells on Day 40, which are present and highly ordered on all myometrial cells on Days 80, 120, and 140 of pregnancy. These highly ordered IACs are not present on the myometrial cells on Day 15 of the estrous cycle or pregnancy and are not present in the myometrium of postpartum ewes. Views of myometrial cells cut in cross-section and in longitudinal section are shown. An irrelevant rabbit IgG served as a negative control. The width of each field is 140 μm.

The temporal and spatial localization of ITGA5 and ITGB1 in the myometrium of pregnant and postpartum sheep were determined by immunofluorescence staining (Figure 3 and Figure 4). Scattered punctate immunostaining for ITGA5 and ITGB1 was detected at the surface of myometrial cells in both longitudinal and circular layers of Day 15 cyclic (shown for ITGB1, Figure 4) and pregnant sheep myometrium (Figure 3 and Figure 4). The punctate staining was more obvious in the Day 15 pregnant myometrium. Longitudinal strands of IACs developed at the surface of the myometrial cells by Day 40 of pregnancy; and increasingly ordered IACs were present in Day 80, Day 120, and Day 140 myometrium (Figure 3 and Figure 4). ITGA5 and ITGB1 continued to be highly expressed in postpartum myometrium although they were no longer organized into longitudinal IACs at the surface of myometrial cells on postpartum Day 1, Day 7, or Day 14 (Figure 3 and Figure 4).

**Figure 5:**
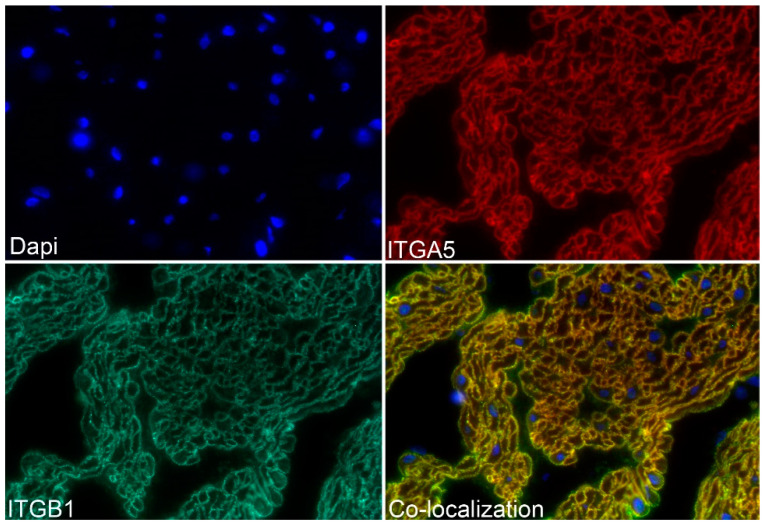
Double-immunostaining for alpha 5 integrin (ITGA5, red color) and beta 1 integrin (ITGB1, green color) in the myometrium of ewes on Day 80 of pregnancy is shown. ITGA5 and ITGB1 precisely co-localized to the same regions on the surface of the myometrial cells (yellow color), confirming the presence of the ITGA5B1 integrin receptor. DAPI was used to stain nuclei for histological reference. The width of each field is 220 μm.

**Figure 6:**
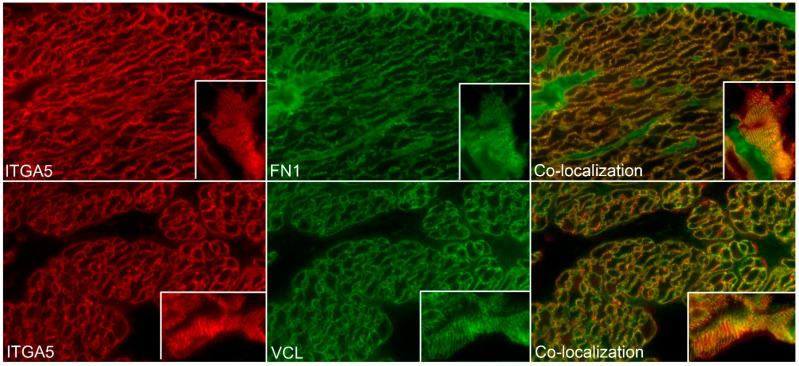
Assembly of integrin adhesion complexes (IACs) in sheep myometrium. Immunostaining for alpha 5 integrin (ITGA5, red color), fibronectin (FN1, green color), and vinculin (VCL, green color) in the myometrium of ewes on Day 80 of pregnancy is shown. ITGA5 and FN1, and ITGA5 and VCL co-distributed to the same regions on the surface of the myometrial cells (note), confirming the presence of IACs. Note that while ITGA5 and VCL are co-localized, VCL is located deeper within the cytoplasm therefore fluorophores do not always overlap. The width of each field is 220 μm; inserts are between 20–30 μm wide.

**Figure 7:**
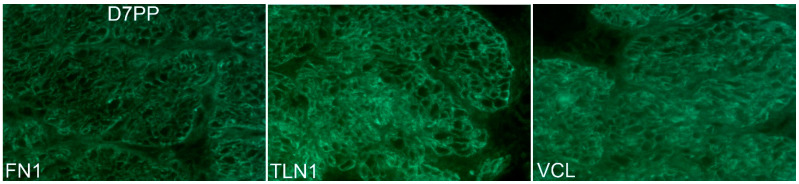
Integrin adhesion complexes (IACs) in sheep myometrium disassemble postpartum. Immunostaining for fibronectin (FN1), talin (TLN1), and vinculin (VCL) in the myometrium of ewes on Day 7 postpartum (D7PP) is shown. Similar to results for the alpha 5 integrin (ITGA5) and the beta 1 integrin (ITGB1) shown in Figure 3 and Figure 4, the multimodular, mechanosensory ECM protein FN1 and intracellular mechanosensory cytoskeletal proteins TLN1 and VCL no longer assemble into IACs in the myometrium of postpartum ewes. The width of each field is 220 μm.

The ITGA5 subunit partners exclusively with the ITGB1 subunit forming the ITGA5B1 receptor [1], and the spatial pattern of expression of ITGA5 and ITGB1 precisely overlapped in the myometrium of Day 80 pregnant ewes, confirming the presence of the ITGA5B1 integrin receptor at the surface of myometrial cells (Figure 5). Further similar spatial immunostaining patterns were observed for the mechanosensory ECM protein FN1 and ITGA5, and for ITGA5 and the intracellular mechanosensory cytoskeletal protein VCL in the myometrium of Day 80 pregnant ewes, confirming the assembly of IACs at the surface of myometrial cells (Figure 6). It is noteworthy that the overlap of expression for ITGA5 with FN1 and VCL was not as exact as the overlap of expression for ITGA5 and ITGB1. ITGA5 and ITGB1 both span the cell membrane. However, FN1 is present external to the cell in the ECM and VCL is located about 40–60 nm deep within the cytoplasm of the cell [27]. Therefore, the expression of FN1 and VCL do not precisely overlap spatially with the expression of ITGA5, and a yellow immunofluorescence signal is not observed. FN1, VCL, and the intracellular mechanosensory cytoskeletal protein TLN1 no longer assembled into IACs in the myometrium of postpartum ewes (Figure 7).

### 3.3. FN1 Stimulates Activation Of ITGA5, ITGB1, and TLN1 to Rapidly Form IACs

**Figure 8:**
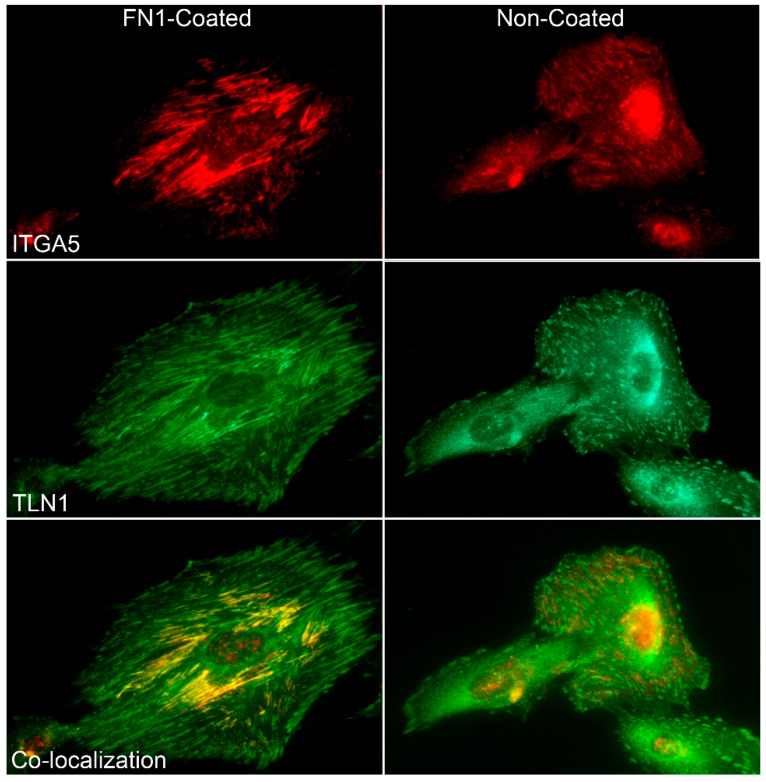
Immunofluorescence localization of the alpha 5 integrin (ITGA5) and talin (TLN1) on human myometrial cells bound to fibronectin (FN1). Cells were seeded on coverglass chambered slides coated with FN1 and immunostained with antibodies to ITGA5 and TLN1. Both ITGA5 and TLN1 were observed in large aggregates as components of integrin adhesion complexes (IACs) at the basal surface of myometrial cells as they attached to the FN1-coated slides. Although cultured cells secrete ECM and bind to that ECM at their basal surface while attaching to slides, note the increased number of IACs for cells attaching to FN1-coated versus non-coated slides. The width of each field is 140 μm.

As a corollary to the increasing order of IACs during expansion of the uterine wall and greater forces applied between FN1, the fibronectin receptor, and cytoskeletal adaptor protein TLN1, we compared the attachment of human myometrial cells to uncoated and FN1-coated culture dishes following the seeding of cells into culture medium containing fetal bovine serum. While serum contains growth and attachment factors and cultured cells secrete ECM, there was an increased spreading of cells, plus the number and size of IACs in cells attaching to FN1-coated slides compared with non-coated slides (Figure 8). Immunostaining of ITGB1 and TLN1 at the basal surface of the cells indicated that rapid in vitro assembly of IACs on a FN1 rigid substrate was occurring (data not shown). Comparable results showing co-distribution of ITGA5 with TLN1 at the basal surface of the cells confirm the rapid in vitro assembly of IACs to FN1 on a rigid substrate (Figure 8).

## 4. Discussion

Constant changes in the lumen diameter of hollow organs generate forces that strain the intercellular connections between smooth muscle cells within the muscularis externa [28]. Cells within tissue-level compartments respond to extracellular mechanical forces by integrin-activated assembly of IACs that dynamically respond to those forces [29,30]. IACs are complex structures composed of transmembrane integrins activated by attachment to ECM proteins that connect to the cytoskeleton and serve as signaling centers that can regulate numerous physiological processes [4]. As external forces sensed through integrins increase, IACs grow and mature, which leads to actin remodeling that transduces intracellular forces necessary to balance changing external forces [29,30]. The myometrium of the pregnant uterus is exposed to external forces that differ over the course of pregnancy from those forces applied to the smooth muscle cells of blood vessels or tubal organs that undergo peristalsis. As pregnancy progresses in women, the uterus can increase 500- to 1000-fold in volume and 24-fold in weight. Therefore, the uterine wall must adapt to increases in fetal growth, placental fluid volumes, blood flow, and hormonal profiles in order to transform it into an organ that can forcefully expel the fetus and placental membranes during parturition [31].

In response to these unique forces in rodents, myometrial cells assemble IACs, but these IACs develop during late pregnancy. Both the hormones of pregnancy and mechanical stretch upregulate the expression of FN1, the integrin receptor ITGA5B1, and other IAC constituents, including the cytoskeletal mechanosensor TLN1 [1,2,32,33]. Both TLN1 and vinculin (VCL) are recognized as cytoplasmic tension-transducing proteins whose signaling activity is modulated by force-induced conformational changes in IACs [34,35]. FN1 is a multimodular force-bearing ECM protein that can exhibit a wide range of conformations in the ECM based upon substrate rigidity [12]. It is secreted by cells and assembled into a matrix that binds to cell surface integrin receptors, which, under tension, can lead to conformational changes of FN1 into different functional signaling states [11,12,13,14]. FN1-mediated focal adhesion kinase activation through the FN1 receptor is dependent on mechanical tension which, in contrast to the collagen I receptor, is decoupled from substrate rigidity or mechanical tension [36,37]. The growth of myometrial IACs is sensitive to the rigidity and strength of adhesion to the ECM [38] and the IAC linkage to the ECM and the myometrial actomyosin complex provides sufficient force to expel the fetus at term [39].

Human fetuses are larger than those of rats, and therefore in women, mRNA and protein for the alpha 1 (ITGA1), alpha 3 (ITGA3), ITGA5, alpha 7 (ITGA7), alpha v (ITGAV), ITGB1, beta 2 (ITGB2), beta 3 (ITGB3), and beta 5 (ITGB5) are significantly higher in term myometrial samples than in nonpregnant control samples [21]. Therefore, it is likely that their presence within the uterus generates more significant mechanical stretch of the uterine wall than rats. The present study is the first to comprehensively examine, across the extent of pregnancy, IAC assembly within the myometrium of a species larger than rats, where the stretch forces applied to the myometrium during gestation are expected to be amplified. Results of the present study indicate that a similar process of IAC assembly takes place in sheep myometrium as occurs in rodents and humans during pregnancy. Interestingly, punctate staining of ITGA5 and ITGB1 subunits was detected in cyclic ewes on Day 15, but punctate staining of these integrin subunits was greater in Day 15 pregnant animals. This punctate staining in cyclic animals has not been reported in rodents. Immunostaining of the integrin subunits increased and was co-distributed with FN1, VCL, and TLN1 on the surface of myometrial cells by Day 40 of pregnancy, indicating the assembly of IACs. Therefore, well-defined IACs begin to assemble in the myometrium of sheep during the first trimester, which is significantly earlier than IACs assemble during pregnancy in rodents. The magnitude and organization of these IACs continue to increase concurrently with the increasing accumulation of fluids in the allantois and amnion and growth of the fetus, suggesting that mechanical stretch of the uterine wall contributes sufficient continuous local force by Day 40 of pregnancy to initiate the development of ordered IACs in the myometrial cells of sheep. Because sheep have a fetus near the size of the human fetus, first trimester assembly of IACs in the myometrium of women may occur in a manner similar to described here for the sheep. This highlights the continued value of the sheep model as a flagship gynecological model for understanding parturition in humans [5]. Further, these IACs disassemble by Day 1 postpartum, although expression of the proteins was maintained for at least 14 days, presumably due to the size of the gravid uterus and duration of uterine involution.

Earlier studies have shown that other tissue compartments within the uteri of domestic species exhibit tissue-specific organization of IACs during pregnancy. First, the subepithelial uterine stromal cells of sheep and pigs differentiate into a myofibroblast phenotype associated with the upregulation of the cytoskeletal proteins desmin, vimentin, and alpha smooth muscle actin to augment the contractility of fibroblasts [40,41]. These stromal cells also express ITGAV and ITGB3 integrins, as well as the ECM proteins secreted phosphoprotein 1 (SPP1, also known as osteopontin (OPN)), FN1, and vitronectin (VTN). The stromal ITGAVB3 receptor binds VTN, FN1, and SPP1 to form IACs, and the diffuse spatial pattern of localization of these proteins within the stroma suggests they are organized into 3D matrix adhesions that developed in a mechanically stressed but more strain-shielded environment [22]. Second, in pigs, it is hypothesized that folding at the interface between the uterine luminal epithelium (LE) and the non-invasive placental chorionic epithelium (CE) is driven by external forces delivered to this interface. Subepithelial endometrial blood vessels deliver increased blood flow that pushes upward on the interface between the uterine LE and the placental CE to trigger focal IAC assembly, and endometrial fibroblasts differentiate into contractile myofibroblasts that pull connective tissue downward and inward to sculpt folds at the uterine-placental interface [41]. Third, it is proposed that SPP1 binds integrin receptors expressed by the uterine LE and placental trophectoderm of both sheep and pigs to form IACs that adhere the uterus to the placenta during the attachment phase of implantation and the development of synepitheliochorial and epitheliochorial placentation, respectively [42,43,44].

In the present study, the ability of myometrial cells to dynamically adapt to changes in extracellular force was also illustrated by the formation of IACs in human myometrial cells on FN1-coated glass chambered slides. Results showed that the ITGA5 integrin subunit was abundantly expressed at the basal surface of cultured myometrial cells and aggregated to IACs at sites of cell anchorage to the substrate. Pre-coating of the substrate with the cell attachment fragment of FN1 enhanced the size and length of in vitro IACs compared to the untreated substrate, illustrating the dynamic ability of cells to respond to ECM stiffness through the FN1 receptor.

## 5. Conclusions

In summary, IACs begin to assemble within the sheep myometrium during early-to-mid gestation in response to increased stretch of the uterine wall and continue to increase as pregnancy progresses. FN1 is essential for IAC assembly and these IACs contribute to a mechanical syncytium that allows for the sensing of mechanical forces from both inside and outside of the cell in order to sustain powerful contractions during labor. After parturition, IACs are disassembled but the integrin subunits ITGA5 and ITGB1 remain expressed at the protein level at least two weeks postpartum, indicating that turnover of these proteins is much slower than their synthesis and suggesting that integrins may contribute to the involution process of an organ capable of remarkable myometrial hypertrophy and hyperplasia. Results of the present study indicate that sheep are similar to humans regarding the assembly of IACs in the pregnant myometrium and suggest that IACs may form much earlier in human gestation than was previously implied by the rat model. Results highlight the continued value of the sheep model as a flagship gynecological model for understanding parturition in humans [5].

## Data Availability

Not applicable.
